# Registry-derived stage (RD-Stage) for capturing cancer stage at diagnosis for endometrial cancer

**DOI:** 10.1186/s12885-023-11615-6

**Published:** 2023-12-12

**Authors:** S. M. Evans, K. Ivanova, R. Rome, D. Cossio, CHC Pilgrim, J. Zalcberg, Y. Antill, L. Blake, A. Du Guesclin, A. Garrett, D. Giffard, N. Golobic, D. Moir, S. Parikh, A. Parisi, K. Sanday, C. Shadbolt, M. Smith, L. Te Marvelde, K. Williams

**Affiliations:** 1 Cancer Council Victoria, Melbourne, Australia; 2 Epworth Health Care, Melbourne, Australia; 3 Cancer Alliance Queensland, Woolloongabba, Australia; 4 Central Clinical School, Department of Surgery, The Alfred, Monash University, Melbourne, Australia; 5 School of Public Health and Preventive Medicine, Monash University, Melbourne, Australia; 6 Monash University, Melbourne, Australia; 7 Department of Anatomical Pathology, The Alfred, Melbourne, Australia; 8 Queensland Centre for Gynaecological Cancer, Royal Brisbane and Women’s Hospital, Brisbane, Queensland Australia; 9 ACT Cancer Registry Australian Capital Territory Health, Deakin, Australia; 10 Royal Women’s Hospital, Melbourne, Australia

**Keywords:** (MeSH Headings) registries, Neoplasm staging, Epidemiology, Cancer, Cancer registries, TNM Stage

## Abstract

**Background:**

Capture of cancer stage at diagnosis is important yet poorly reported by health services to population-based cancer registries. In this paper we describe current completeness of stage information for endometrial cancer available in Australian cancer registries; and develop and validate a set of rules to enable cancer registry medical coders to calculate stage using data available to them (registry-derived stage or ‘RD-Stage’).

**Methodology:**

Rules for deriving RD-stage (Endometrial carcinoma) were developed using the American Joint Commission on Cancer (AJCC) TNM (tumour, nodes, metastasis) Staging System (8^th^ Edition). An expert working group comprising cancer specialists responsible for delivering cancer care, epidemiologists and medical coders reviewed and endorsed the rules. Baseline completeness of data fields required to calculate RD-Stage, and calculation of the proportion of cases for whom an RD stage could be assigned, was assessed across each Australian jurisdiction. RD-Stage (Endometrial cancer) was calculated by Victorian Cancer Registry (VCR) medical coders and compared with clinical stage recorded by the patient’s treating clinician and captured in the National Gynae-Oncology Registry (NGOR).

**Results:**

The necessary data completeness level for calculating RD-Stage (Endometrial carcinoma) across various Australian jurisdictions varied from 0 to 89%. Three jurisdictions captured degree of spread of cancer, rendering RD-Stage unable to be calculated. RD-Stage (Endometrial carcinoma) could not be derived for 64/485 (13%) cases and was not captured for 44/485 (9%) cases in NGOR. At stage category level (I, II, III, IV), there was concordance between RD-Stage and NGOR captured stage in 393/410 (96%) of cases (95.8%, Kendall’s coefficient = 0.95).

**Conclusion:**

A lack of consistency in data captured by, and data sources reporting to, population-based cancer registries meant that it was not possible to provide national endometrial carcinoma stage data at diagnosis. In a sample of Victorian cases, where surgical pathology was available, there was very good concordance between RD-Stage (Endometrial carcinoma) and clinician-recorded stage data available from NGOR. RD-Stage offers promise in capturing endometrial cancer stage at diagnosis for population epidemiological purposes when it is not provided by health services, but requires more extensive validation.

**Supplementary Information:**

The online version contains supplementary material available at 10.1186/s12885-023-11615-6.

## Background

Cancer stage at diagnosis is an important data field to be captured by population-based cancer registries (“cancer registries”) and by services delivering clinical care. For solid tumours, it categorises the size, location, and extent of the primary tumour and whether it has infiltrated lymph nodes and/or distant organs.

Cancer staging is used to forecast cancer prognosis, evaluate the efficacy and effectiveness of cancer screening programs [1], determine the most appropriate treatment for patients diagnosed with cancer, identify potentially suitable participants for clinical trials and evaluate the impact of treatments and clinical trials on cancer survival, recurrence, and treatment response [2]. Stratifying treatment and outcomes by cancer stage provides an important tool to examine inequities in access to cancer-related services and care [3]. More recently, cancer stage data has been used to assess the impact of COVID-19 pandemic related disruptions to curative-intent treatment on survival [4]. Because advanced cancer stage at diagnosis usually incurs an additional treatment burden to patients while early-stage disease requires only localized treatment, cancer stage data is considered one of the most important cancer data elements to project the economic burden of cancer and forecast cancer service needs, such as for survivorship services and where radiotherapy/outreach programs are required [5].

There are three approaches to staging cancers. The most commonly used approach stages cancers using the TNM Staging System (8^th^ Edition), developed and maintained by the American Joint Committee on Cancer (AJCC) and the Union for International Cancer Control (UICC) [6]. There are iterations of the TNM staging system, such as the Condensed TNM and Essential TNM, used by cancer registries in the absence of any or all TNM data elements [7]. Another approach to staging cancer uses categories of localised, regional, or distant spread. An example of this is the Degree of Spread staging system adopted by some Australian cancer registries [8]. The third approach uses disease-specific staging systems. An example of this is the International Federation of Gynecology and Obstetrics (FIGO) staging system used for gynaecological tumours [9]. The FIGO staging system is preferred by specialists treating patients with endometrial cancer, because it uses specific criteria tailored to gynaecological tumours and can be directly translated to the TNM staging system.

Despite its widespread utility, cancer stage information is poorly captured by many cancer registries. Stage is often not recorded in machine-readable structured fields in electronic medical records, so it is not easily captured by medical coders and transmitted to the registry. Reasons for poor reporting of stage likely include user and system attributes, lack of support needs, clinical workflow issues, and environmental factors [10, 11]. Even when a targeted intervention was undertaken using email reminders to oncologists prompting the recording of stage in the electronic medical record, at its highest point in the intervention only 40% of new cancer patients had stage of disease documented at diagnosis [12]. A US study exploring the completeness of stage data for colorectal cancer patients found a similar rate (38% provided complete TNM stage data in the electronic medical record), although this increased to 73% when any clinical notation of stage was accepted [13].

Routine collection of cancer stage at diagnosis is an identified national data gap in our cancer knowledge. Since 2014, Cancer Australia, through the Stage, Treatment and Recurrence (STaR) initiative, has led work to progress opportunities for the collection, access and transfer of cancer data on stage, treatment and recurrence for a range of cancers [14]. Through collaboration with state and territory cancer registries, the Australasian Association of Cancer Registries (AACR) and state and territory health departments, this work led to AACR-endorsed rules for collecting registry-derived (RD) stage for five tumour types. RD-Stage was defined as “*the best estimate of summary TNM stage at the time of diagnosis (or within 120 days of diagnosis and before primary cancer treatment) as derived by cancer registries from available data sources for use in population data analysis”* [15]*.* Available data sources included pathology reports and data provided by hospital administrative systems. Because RD-Stage is calculated using only this minimal data and does not consider other inputs such as imaging scans and clinical examination to determine stage, it was intended for epidemiological population-based analyses only and not to be used at a clinical level for individual patients [16].

RD-stage rules for breast, prostate, lung, and bowel cancer and melanoma have been validated against stage data collected in New South Wales and South Australia. In New South Wales, comparison against stage captured through manual record review demonstrated good to excellent concordance [17]. In South Australia, RD-stage was compared with pathology data and stage recorded in a clinical registry (which used the full medical record as the source of information about the cancer case) for breast and bowel cancer. RD-Stage resulted in a higher level of completeness of stage and good to excellent agreement compared to both pathology and clinical registry data [18]. This work led to publication of the first national stage at diagnosis data for the five highest incident cancers in 2011 (breast among females, colorectal, lung and prostate cancers and melanoma) [19] and ultimately 5-year survival outcomes (to 2016) for this cohort [20]. RD-Stage is routinely reported as an annual trend by the Victorian Cancer Registry (VCR) for bowel and breast cancer, and melanoma [21]. RD-stage (Lung cancer) is not routinely reported by the VCR because of poor completeness at a population-level (~ 57%) and RD-Stage (Prostate cancer) is not reported as it has not been updated to reflect changes outlined in the 8^th^ edition of the AJCC staging manual.

Given the ongoing challenges of capturing cancer stage at diagnosis from medical records, the demonstrated utility of RD-Stage and continuing the work of the STaR initiative, Cancer Australia supported the development of further RD-Stage rules. Endometrial cancer was selected because it is among the most commonly diagnosed cancers in females (5^th^ most common in Victoria in 2021) [22], and the vast majority of new diagnoses have accompanying surgical pathology. Cancer staging for endometrial cancer relies on surgical pathology rather than diagnostic biopsy or curettage specimens due to its comprehensive evaluation of tumour size, invasion depth, lymph node involvement, margin assessment and tumour grading. In this paper we (1) describe the process taken to develop RD-Stage (Endometrial carcinoma); (2) provide details of the baseline capacity of cancer registries to report RD-Stage (Endometrial carcinoma); and (3) outline the results of a validation study to assess the concordance of RD-Stage (Endometrial carcinoma) with stage reported in clinical notes.

## Methods

This study was undertaken in Victoria, Australia between July 2021 and September 2022.

### Development of staging rules

Rules for staging endometrial cancer by medical coders in cancer registries were developed using the TNM Staging System (8^th^ Edition) [6]. An expert working group was assembled comprising epidemiologists (*n* = 2), clinical coding consultants (*n* = 4), a radiologist, consultant pathologists (*n* = 2), a medical oncologist, consultant gynaecological oncologists (*n* = 2) and a statistical analyst. Draft rules were written by KI and distributed in advance of the working group meeting with instruction to review the histology codes to be included in the model, assess the rules for each tumour (T)-, node (N)- and metastases (M)- category, determine the time point up to which diagnostic stage can be measured and review diagnostic and treatment pathways used to assist medical coders in coding and following up on potentially missing information. The meeting was held via videoconference over two hours, with correspondence occurring via email after the meeting. A second meeting discussed unresolved issues and reached consensus on the rules.

In reviewing the initial draft RD-Stage rules, the expert working group recommended that evaluation and validation of uterine malignancy be restricted to carcinoma of the endometrium (International Classification of Diseases for Oncology version 3 (ICD-O3 [23]) topography code C54.1) as this constituted the largest subgroup of uterine malignancies (> 90%) and rules for other subtypes of uterine malignancies differed markedly from endometrial carcinomas. Further to this, they noted that survival rates differed between endometrial and non-endometrial malignancies of the uterus and that the small number of non-endometrial uterine malignancies meant it would take many years for smaller states to acquire sufficient cases to provide meaningful analyses of stage data. A list of eligible histology codes for which RD-Stage (Endometrial carcinoma) can be calculated and rules are provided in Supplementary material 1 and the normal diagnostic and treatment pathway for endometrial cancer is outlined in Supplementary material 2.

The working group recommended that RD-Stage calculation commence with calculating the TNM-M value, as the presence of metastases negated the need to capture the TNM-T or TNM-N category. There was debate over whether M0 could be assumed if it was not explicitly stated as such, and in the absence of M1 being recorded. Classification of MX (unknown) was eliminated from the AJCC and UICC TNM staging systems in the 6^th^ edition. Current rules state that “Unless there is clinical or pathological evidence of distant metastases, the patient should be classified as clinical M0 and denoted as clinical M0 (cM0). A history and physical examination are all that is needed to assign cM0. The M category must always be known and reported to assign a stage group” [6]. Queensland modified the assumption of M0 by requiring a negative metastatic coding in a subsequent hospital admission more than 120 days after the date of diagnosis to record an M0 status. If no hospital admission occurred after the initial diagnostic admission, then M0 is not assumed in Queensland patients. Because cancer registries do not routinely have access to all hospital admission data, these rules could not be tested in other jurisdictions. RD-Stage (Endometrial carcinoma) groups derived from the assigned TNM-T, TNM-N and TNM-M categories are shown in Table 1.

**Table 1:** RD-Stage derivation from assigned TNM-T, TNM-N, and TNM-M categories for endometrial cancer

RD Stage group	RD-Stage sub-categories	FIGO 2009	T	N	M
1	1	I	T1	N0,	M0
	1A	IA	T1a	N0	M0
	1B	IB	T1b	N0	M0
2	2	II	T2	N0	M0
3	3	III	T3	N0	M0
	3A	IIIA	T3a	N0	M0
	3B	IIIB	T3b	N0	M0
	3C1	IIIC1	Tx, T1-T3	N1,	M0
	3C2	IIIC2	Tx, T1-T3	N2,	M0
4	4A	IVA	T4	Any N	M0
	4B	IVB	Any T	Any N	M1
Unknown	9		TX	N0, X	M0

### Baseline capture of endometrial cancer stage at diagnosis by cancer registries

Following the development and endorsement of RD-Stage (Endometrial carcinoma) rules, cancer registries were requested to complete an Excel spreadsheet containing the data fields required to calculate RD-Stage for the years 2018–2019, to ascertain a baseline completeness of RD-Stage for endometrial cancer. As three jurisdictions (New South Wales, Tasmania, and Australian Capital Territory) routinely captured Degree of Spread, this information was requested so that baseline completeness of both Degree of Spread and RD-Stage could be assessed and compared. Queensland used a broader range of data sources via the Cancer Alliance Queensland’s data holdings to capture RD Stage, which included oncology information systems, multidisciplinary meeting discussion, hospital admitted episode data and public radiology reports.

### Validation of RD Stage (Endometrial carcinoma) rules

To test the RD-Stage rules, a validation dataset was obtained from the National Gynae-Oncology Registry (NGOR). The NGOR was established in 2018 as a clinical quality registry, in accordance with the strategic principles outlined in the Australian Commission on Safety and Quality in Health Care’s framework for clinical quality registries [24]. It is operated by the Cancer Research Program in the School of Public Health and Preventive Medicine at Monash University [25]. Recruitment of patients to NGOR has occurred progressively, as ethical approval is obtained from recruiting hospitals. At the time of this study, seven Victorian hospitals were recruiting patients [26]. NGOR data are captured either (1) directly by the patient’s treating physician entering information into their respective data systems, from which an extract is forwarded to Monash University for uploaded into NGOR; or (2) by trained research assistants abstracting the information from the patient’s medical record. NGOR research assistants were directed to only record stage if it was expressly recorded in the patient’s medical record.

Two medical coders were trained in abstracting information for RD-Stage calculation from surgical pathology reports submitted to the VCR. Data was populated into an Excel spreadsheet in which a macro was written to automatically calculate RD-Stage. Coders entered data for cases with eligible histology codes diagnosed between 2018 and 2020.

A file containing patient details and pathological and clinical stage information for cases diagnosed between 2018 and 2020 was provided by NGOR, where it was linked with cases from the VCR. Deterministic matching was used to link the datasets, using first name, middle name (where present), last name, Medicare number and date of birth.

A manual medical record review was undertaken to independently assess the accuracy of stage data captured by NGOR. Due to COVID-19 pandemic-related restrictions, the medical record review could only be undertaken in one health service. A blinded sample of 5% of all concordant cases at this hospital, and review of all discordant cases was undertaken by a medical coding expert (KI).

## Results

### Baseline capture of endometrial cancer stage at diagnosis by cancer registries

Table 2 provides an outline of the completeness of data fields required to calculate RD-stage across Australian jurisdictions in 2018 and 2019. Because three jurisdictions used only Degree of Spread stage classification, RD-Stage (Endometrial carcinoma) could only be calculated in Victoria, Queensland, South Australia, Western Australia, and Northern Territory. All jurisdictions capable of calculating RD-Stage had access to pathology data, yet only Victoria and Queensland had routine access to hospital admitted episode data to enable metastatic site at diagnosis ICD-10 C77-79 codes to be captured. Western Australia reported capturing stage details for one small, targeted study and Tasmania reported only capturing stage group if it was expressly recorded by the hospital or on the pathology report. South Australia captured no stage data for either year. The wide variety of data systems in Queensland, from which stage data could be obtained, led to the highest level of stage completeness reported in this jurisdiction.

**Table 2:** Completeness of selected data fields at baseline for endometrial cancer, by jurisdiction, 2018 and 2019 combined

Jurisdiction	VIC	QLD	WA	SA	NSW	TAS	ACT	NT
Number of cases (n)	1459	1125	496	480	1757	71	102	32
Data completeness								
Nodes taken	43%	53%	-	-	-	22%	-	-
Nodes positive	43%	6%	-	-	-	22%	-	-
Staging basis	88%	90%	6%	-	-	-	-	9%
TNM-T	84%	58%	2%	-	-	-	-	25%
TNM-N	44%	43%	1%	-	-	-	-	6%
TNM-M	5%	29%	-	-	-	-	-	-
Stage group	15%	25%	5%	-	-	-	-	16%
Degree of spread (Tier 1)^a^	-	-	-	-	93%	84%	98%	-
RD-Stage (Tier 2)^b^	88%	37%	6%	-	-	-	-	25%
RD-Stage (Tier 3)^c^	-	90%	-	-	-	-	-	-

^a^Degree of spread (Tier 1) data sources: Pathology and hospital admitted episode data (including access to metastatic site codes (NSW, ACT, TAS) and clinical medical records (TAS and NSW)

^b^RD-Stage (Tier 2) data sources: Pathology (VIC, WA, NT, QLD) and hospital admitted episode data (VIC)

^c^RD-Stage (Tier 3) data sources: In addition to Tier 2, MDM data, hospital admitted episode data oncology information systems and public radiology data (QLD)

### Validation of RD-stage

For the 2018–2020 years, there were 2020 newly diagnosed cases of endometrial cancer recorded in the VCR, of which 485 patient records (24%) were captured in the NGOR database and were linked to the VCR. However, of these 485 cases, 36 had stage unknown from both datasets, stage was unknown in both datasets 21 unique cases were without TNM stage reported by the VCR and 12 were without TNM stage reported in the NGOR. There were 6 cases on NGOR which were deemed ineligible because they contained morphology codes not permitted or were identified as not endometrial by site of origin. After exclusion criteria were applied, direct comparison of stage was available for 410/485 (85%) of cases (Table 3).

**Table 3:** Stage distribution for cases reported in the Victorian Cancer Registry (VCR) and the National Gynae-Oncology Registry (NGOR), 2018–2020

	**VCR-RD stage**	**NGOR**
Stage 1	310 (64%)	323 (67%)
Stage 2	28 (6%)	31 (6%)
Stage 3	62 (13%)	57 (12%)
Stage 4	21 (4%)	30 (6%)
Stage Unknown	64 (13%)	44 (9%)
TOTAL	485	485
*Excluded from staging validations* • *Stage unknown in VCR (n* = *21)* • *Stage unknown in NGOR (n* = *12)* • *Stage unknown in both (n* = *36)* • *Ineligible morphology code (n* = *6)*		
Total number of patients for Stage Validation (n)	410/485 (85% of cases)	

In total, 17 non-concordant cases were identified at a category level (stage I-IV). There were a further 13 cases which were non-concordant at sub-category level (e.g. Stage IIIA vs IIIB). At the stage category level, there was agreement in stage for 393/410 cases (95.8%) with Kendall’s coefficient of concordance of 0.95 indicating a very good level of agreement (Table 4) [27].

**Table 4:**
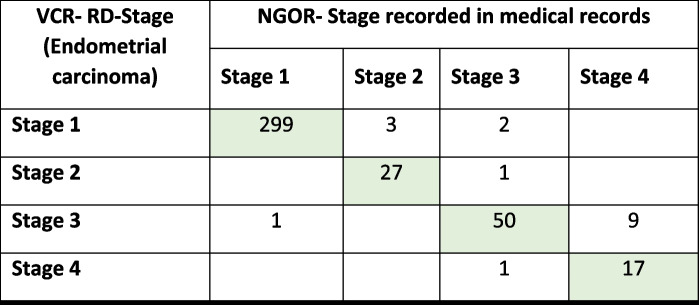
Distribution of stage in the Victorian Cancer Registry (VCR) and National Gynae-Oncology Registry (NGOR), 2018–2020

Agreement = 393/410 (95.8%) Kendall’s coefficient of concordance = 0.95

In total, 224/485 (55%) cases reported by NGOR were from the site for which an audit could be undertaken. The random audit of 5% of concordant cases identified no issues. Review of cases where there was discordance at a category level resulted in VCR upgrading two cases from in situ to invasive based on clinical diagnosis. VCR was missing pathology reports for seven cases and recorded unknown for 10 cases for which NGOR was able to use clinical information to capture stage. Three cases were incorrectly staged by VCR medical coders despite the availability of staging information. In one of these cases, the coder missed an Addendum report changing the diagnosis from urothelial carcinoma to endometrioid carcinoma. In the second case, a VCR coder incorrectly recorded TNM *N* = 1 based on presence of isolated tumour cells (ITC) in a pelvic regional node. In the third case, a VCR coder missed reporting mesenteric metastases. VCR updated the tumour and staging data for these three cases.

The audit identified that NGOR was missing pathology reports for eight cases, misclassified four cases as endometrial cancer and reported stage outside the diagnostic period for two cases. Four cases were incorrectly staged by NGOR. Two of the cases were staged as stage 2 (cervical stromal invasion), despite the pathology reports and doctor’s correspondence indicated stage 1A. The third case was staged as 1B, based on myometrial depth of invasion reported as 2/12mm (stage 1A). The stage 4B in the fourth case was based on distant metastases originated from a small bowel neuroendocrine tumour.

## Discussion

Hospitals and pathology providers across all Australian jurisdictions poorly report stage of endometrial cancer at diagnosis to cancer registries. Cancer stage at diagnosis for patients diagnosed with endometrial cancer is best captured in Queensland, where it is expressly documented in pathology reports in 25% of cases. With access to a suite of other clinical datasets it increased to 90%. Similarly, despite very poor reporting of stage in Victoria (15% of cases had stage reported), use of RD-Stage rules enabled 88% of cases to record stage at diagnosis.

In other jurisdictions, stage is neither explicitly reported nor able to be derived using the RD-Stage rules in most cases. The heterogeneity in datasets used, and data elements captured, by each cancer registry provides a significant barrier to reporting stage in a consistent manner at a national level. While hospitals in Victoria are mandated to report nodal and metastatic sites (range C77-C79 in the International Classification of Diseases 10th edition (ICD-10) to the VCR [28], thereby populating the TNM-M data field, this is not a required field in other jurisdictions despite it being captured on all inpatients in hospitals at a national level. While the lack of inpatient data capturing metastatic disease for endometrial cancer only has a minimal impact on the capacity of cancer registries to report stage at diagnosis, because few cases are diagnosed with metastatic disease, for other tumour groups such as for pancreatic and lung cancer, where nearly 50% of patients are diagnosed with metastatic disease [29], the addition of these data fields would significantly improve reporting of stage at diagnosis and improve capacity to assess health system performance. The implication of these findings is that, where stage is not explicitly reported to cancer registries by health services, investment in medical coders and in transmission of admitted episode data by hospitals, will enable RD-Stage to be reported by cancer registries for most patients to a standard comparable with clinical stage reported by clinicians.

The use of rules to assume M0 played an important part in classifying stage, because M0 was explicitly reported in less than 1% of non-metastatic cancer notifications. Queensland was able to capture stage from a wider range of data sources and as such, modified the rules to not assume M0 disease. However, it was reassuring to see a high level of concordance between stage captured using the rules and clinical stage reported in the clinical quality registry, NGOR.

Other countries have struggled with capturing accurate stage data in their cancer registries. In the United Kingdom, efforts such as the Cancer Waiting Times Initiative have enforced targets and penalties to ensure timely cancer diagnosis and treatment. This has significantly improved the documentation of cancer stage, with stage at diagnosis completion rates of between 71–90% across 18 common cancers being achieved [30].

The Essential TNM staging system was developed using a similar approach to RD-Stage, for under-resourced registries without access to detailed medical records [31]. Rules were developed for breast, cervical, colorectal, liver, oesophageal, ovarian and prostate cancer and lymphoma, categorising tumours into stages I, II, III, and IV using the TNM classification system [32]. A validation study across 20 African countries compared Essential TNM stage assignments made by local coders with those by expert clinicians. The results showed moderate to substantial agreement, though there were challenges due to differences in interpreting clinical terms and rules [33]. The disparities in agreement levels between this study and our study may reflect differences in the quality of information accessible to support coders in capturing RD-stage, as well as the extent and nature of the training they received. Unlike the African study, where coders were spread across 51 sites and underwent a three-day online training course, our medical coders were centrally located in the statewide registry and received continuous face-to-face training support.

Degree of spread is used in New South Wales, Tasmania, and the Australian Capital Territory, and we found that it was well captured by cancer registries for endometrial cancer. Yet, previous validation work has demonstrated that RD-Stage provided at least comparable or more complete and concordant stage information than degree of spread for the five tumours examined [17]. There is growing recognition of the need to have a single, simple, robust cancer staging system to enable international benchmarking to be undertaken; and inability of the localized, regional and metastatic staging system to convert to TNM, and the popularity of the TNM staging system among clinicians and researchers [34], will likely result in its phasing out over time.

The availability of multidisciplinary team meeting, imaging and oncology system data in Queensland enabled stage completeness to be the highest among jurisdictions able to calculate RD-Stage. The RD-Stage rules were only applied when no documented stage was found in these datasets. This approach provided an efficient way of collecting trusted stage, while minimising the effort required by cancer registry staff. This provides important insight into the benefit which would be realised if jurisdictions could incorporate other data feeds and use RD-stage as a supplementary process.

Staging of cancer relies on multiple inputs, such as clinical examination, blood and tissue pathology, and imaging. Our finding that RD-Stage (Endometrial carcinoma) was unable to classify more than 10% of endometrial cancers indicates that either (1) surgery is not performed at diagnosis or as first line treatment; (2) a hospital notification has been received with no accompanying histological specimen and no indication of metastatic disease recorded in administrative data, or (3) the surgery was performed outside the diagnostic period of 120 days. While there may be some under-reporting of metastatic disease by hospital coders, it is likely that most of these unclassified endometrial cancers are localised cancers for which pathology was either not performed or was not transmitted to the cancer registry. The use of automated transmission of pathology data to cancer registries, such as that used in Queensland [35], New South Wales [36] and Victoria [37] has been shown to improve cancer notification by pathology providers. However, this validation study identified that there remain gaps in the provision of pathology reports to cancer registries which need to be addressed. In addition to ensuring laboratories notify all in-scope cancers to cancer registries, it is important that cancer stage is routinely reported in accordance with international and local guidelines. Since 2013, the International Collaboration on Cancer Reporting has included cancer stage as a required field in the endometrial cancer pathology minimum dataset [38]. Now in its 4^th^ edition [39], the Royal College of Pathologists strongly endorses and cross references its protocols to the International Collaboration on Cancer Reporting dataset [40]. Effective from August 2022, the National Pathology Accreditation Advisory Council have advised that for laboratories to be accredited in Australia, the content and format for cancer reporting must be in accordance with the National Structured Pathology Reporting Protocols [41]. Accreditation is required for pathology services to be eligible for Medicare rebates, so this will likely provide a strong lever to improve the reporting of stage on pathology reports [42].

With imaging playing an increasingly important role in diagnosing and staging cancer [43], it is likely that generation of RD-Stage would be enhanced if stage was also reported by imaging services (ideally in a structured format as is required for pathology reporting) or captured well in multidisciplinary team meeting software and made available to cancer registries.

This project has been developed with input from all clinical disciplines involved in the staging of endometrial cancer and from epidemiologists and coding experts. Rules were validated on a sample of 410 cases from a diverse population. However, despite these strengths, there are several noteworthy limitations. RD-Stage (Endometrial carcinoma) rules were developed based on the 8^th^ edition of the AJCC Staging Manual. As staging rules change, so too the RD-Stage rules must be updated. This is evident in prostate cancer, where RD-Stage rules were developed using the 7th edition of the AJCC Staging Manual and have not yet been updated to the 8th edition. Validation of the RD-Stage (Endometrial carcinoma) rules was undertaken using Victorian data provided by NGOR. While it is unlikely that stage is recorded differently in other jurisdictions, local validation is advised if RD-Stage is to be implemented outside Victoria. The quality of recording of RD-Stage depends on the skill of the medical coders. In this study, medical coders responsible for recording RD-Stage were provided with specialised training to assist in interpreting pathology reports. The absence of this training will likely impact the quality of RD-Stage reported.

## Conclusion

The Cancer Australia Australian Cancer Plan aims to make a significant impact on reducing inequities in cancer outcomes over the coming ten years [44]. Investment in a national strategy to improve data infrastructure and standardise data fields captured across jurisdictional cancer registries, provide important building blocks to enable national measurement of the impact of strategies to bridge this gap. The limited validation work undertaken in this project demonstrated that RD-Stage groups compared well with stage reported by clinicians, providing promise that it might be possible to use it at a population-level to stage endometrial cancer when stage group is not reported to cancer registries by hospitals and pathology providers.

### Supplementary Information

**Addditional file 1: Supplementary material 1.**  Staging rules for endometrial cancer. **Supplementary material 2.**  Diagnostic and treatment pathway- endometrial cancer.

## Data Availability

The datasets used and/or analysed during the current study available from the corresponding author on reasonable request
