# Supplementary material for: Registry-derived stage (RD-Stage) for capturing cancer stage at diagnosis for endometrial cancer
Source: BMC Cancer. 2023 Dec 12;23:1222. doi: 10.1186/s12885-023-11615-6 (PMC10714535; doi:10.1186/s12885-023-11615-6)

# Supplementary material 1. Staging rules for endometrial cancer

**HISTOLOGY**

All terms describe malignant tumours, as denoted by the /3 appended to the 4-digit histology codes e.g. 8013/3. ICD-O3 histology codes eligible for staging under the Uterine cancer TNM staging classification:

| **Code** | **Description** |
| --- | --- |
| **8013/3** | Large cell neuroendocrine carcinoma |
| **8020/3** | Undifferentiated carcinoma |
| **8041/3** | Small cell neuroendocrine carcinoma |
| **8070/3** | Squamous cell carcinoma |
| **8240/3** | Carcinoid tumour |
| **8263/3** | Endometrioid carcinoma, villoglandular |
| **8310/3** | Clear cell carcinoma |
| **8323/3** | Mixed cell adenocarcinoma |
| **8380/3** | Endometrioid carcinoma |
| **8382/3** | Endometrioid carcinoma, secretory |
| **8441/3** | Serous carcinoma |
| **8480/3** | Mucinous carcinoma |
| **8570/3** | Endometrioid carcinoma, squamous differentiation |
| **8980/3** | Carcinosarcoma |
| *8000/3* | *Neoplasm, malignant* |
| *8010/3* | *Carcinoma, NOS* |
| *8140/3* | *Adenocarcinoma, NOS* |
| *8255/3* | *Adenocarcinoma with mixed subtypes* |
| *8460/3* | *Papillary serous cystadenocarcinoma* |
| *8461/3* | *Serous surface papillary carcinoma* |
| *8560/3* | *Adenosquamous carcinoma* |
| *8950/3* | *Mullerian mixed tumour (obs)* |
| *in italics: unspecified or outdated morphology* | |

**RULES FOR ASSIGNING TNM-M**

The most common sites of distant metastases for endometrial cancer are bone, lung, liver, intraperitoneal disease, and non-regional lymph nodes.

A positive peritoneal cytology is not included in the AJCC 7th and 8th editions for uterine cancer and does not alter the tumour stage. Therefore, the peritoneal cytology should not be used to code distant metastases.

Metastases to vagina (T3b), adnexa (T3a) and pelvic organs and tissues (T3b) are not considered as distant metastases.

Rules for assigning the M category for endometrial cancers are outlined below.

Rules for coding M- categories for endometrial tumours

| **Value** | **Description** | **Rules** | **RD-Stage** | **FIGO stage** |
| --- | --- | --- | --- | --- |
| **0** | No clinical or imaging evidence of distant metastases | Use “0” in cases with:   - no distant metastasis detected and reported - M=0 reported on medical records - unknown, distant metastasis not state | Record TNM-M=0 and continue to TNM-N |  |
| **1** | Distant metastases as determined by classic clinical, and imaging means and/or histologically proven | Use “1” in any of the following cases:   - reported metastases to non-regional lymph nodes (eg inguinal lymph nodes) - reported distant metastases (excluding metastases to vagina, pelvis, or adnexa) - presence of intraperitoneal disease (eg omental metastases) - reported carcinomatosis | TNM M=1  and  **RD-Stage=4**  (Staging completed) | **IVB**  (Staging completed) |

**RULES FOR ASSIGNING TNM-N**

Regional and non-regional lymph nodes reported for endometrial cancer are outlined below.

Regional and non-regional lymph nodes) for endometrial cancer

| Lymph node site | ICD-10 code | Defines TNM | FIGO stage |
| --- | --- | --- | --- |
| **Pelvic lymph nodes, NOS**:   - Iliac, NOS   - Common iliac   - External iliac   - Internal iliac (hypogastric) - Parametrial - Paracervical - Sacral, NOS   - Lateral (laterosacral)   - Middle (promontorial; Gerota’s node)   - Presacral   - Uterosacral - Obturator | C775 | TNM N=1  Stage 3C1 | IIIC1 |
| **Aortic, NOS**   - Para-aortic | C772 | TNM N=2  Stage 3C2 | IIIC2 |
| **Inguinal lymph nodes, NOS**:   - Deep inguinal (Node of Cloquet or Rosenmuller) - Superficial inguinal - Lymph nodes of groin | C774 | TNM M=1  Stage 4B | IVB |

The following rules should be used when assigning an N category:

1. Sentinel lymph nodes procedure can be considered for patients with apparent uterine-confined endometrial cancer when there is no metastasis found on imaging.
2. Isolated tumour cells (ITC) are defined as single tumour cells or small clusters not greater than 0.2 mm, usually detected only by immunohistochemical (IHC) or molecular methods but which may be verified on hematoxylin and eosin (H and E) stains. ITCs do not usually show evidence of malignant activity (e.g., proliferation or stromal reaction). Lymph nodes with ITCs only are not considered positive lymph nodes.
3. Micrometastases are defined as tumour deposits greater than 0.2 millimetre (mm) but not greater than 2.0 mm in largest dimension. All nodes with micrometastases are included in the count of positive lymph nodes.
4. Macrometastasis are tumour deposits greater than 2.0 mm.
5. For the purposes of the RD-stage project a simplified version of TNM N categories to be used, where the subcategories of TNM N 1mi, 1a, 2mi and 2a values to be collapsed to the main categories TNM -N=1 (metastases to pelvic lymph nodes) and TNM N=2 (metastases to para- aortic lymph nodes).
6. Metastases to the regional nodes on imaging. Assessment of lymph node metastases on imaging is based on:
   - CT and MRI studies: lymph nodes with size >1 cm in the short axial dimension, round shape; presence of central necrosis and heterogeneous or high enhancement, are considered abnormal
   - PET/CT scan: metabolically active lymph nodes of any size are considered abnormal.

Rules for assigning the N category for endometrial cancers are outlined below.

Rules for coding N- categories for endometrial tumours

| **Value** | **Description** | **Rules** | **Staging basis** | **RD-Stage** | **FIGO stage** |
| --- | --- | --- | --- | --- | --- |
| **X** | Regional lymph nodes not assessed | Use “X” in cases with:   - Unknown whether regional lymph nodes were assessed - Regional lymph nodes not assessed | Clinical | Record TNM-N=X and continue to  TNM-T |  |
| **0** | No regional lymph node metastases | Use “0” in cases with:   - reported N=0 - no metastasis identified in regional lymph nodes - reported isolated tumour cells (ITC) without further nodal metastasis on histology | Clinical/ Pathological | Record TNM-N=0 and continue to  TNM-T |  |
| **1** | Regional metastases to **pelvic** lymph nodes (C775) | Use “1” in cases with reported:   - N=1, N1mi or N1a - nodal metastases as C775 on hospital notification without corresponding pathology - involved pelvic lymph nodes on imaging and/or pathology | Clinical/  Pathological | **RD-Stage=3C1**  (Staging completed) | **IIIC1**  (Staging completed) |
| **2** | Metastases to **para-aortic** regional lymph nodes (C772), with or without positive pelvic lymph nodes | Use”2” in cases with reported:   - N2, N2mi or N2a on medical records - Nodal metastases as C772 on hospital notification without corresponding pathology - involved regional para-aortic lymph nodes on imaging and /or pathology | Clinical/  Pathological | **RD-Stage=3C1**  (Staging completed) | **IIIC2**  (Staging completed) |

**RULES FOR ASSIGNING TNM-T**

Rules for classifying T-categories are described below. A positive peritoneal cytology does not impact the TNM T - value since implementation of 7^th^ Ed of AJCC and FIGO 2009 staging classification.

*Rules for coding T- categories for endometrial tumours*

| **Value** | **Description** | **Rules** | **Staging basis** | **RD-Stage** | **FIGO stage** |
| --- | --- | --- | --- | --- | --- |
| **X** | Unknown  Primary tumour not assessed | Use “X” in cases with:   - no information on primary tumour is available - evidence of primary tumour, but no tumour extension reported on histology | Clinical/  Pathological | Record TNM-T=X and continue to Table 16 to derive RD-Stage |  |
| **0** | No evidence of primary tumour | Use “0” in cases with:   - no evidence of primary tumour on pathology   Use “yp0” in case of complete response to neoadjuvant therapy (eg- no residual tumour after treatment) | Pathological | Record TNM-T=0 and continue to Table 16 to derive RD-Stage |  |
| **1** | Tumour confined to corpus uteri, including the endocervical glandular involvement | Use “1” in case of:   - reported as T=1 - reported on histology as: - endocervical glandular invasion only - invasion of myometrium, NOS - localised endometrial tumour | Clinical/  Pathological | Record TNM-T=1 and continue to Table 16 to derive RD-Stage | I |
| **1a** | Tumour limited to the endometrium OR invading < 50% of the myometrium | Use “1a” in case of:   - reported as confined to the endometrium - reported as invasion of inner half of myometrium - endocervical glandular invasion WITH invasion up to 50% of the myometrium - reported as invading less than 50% of the myometrium - detected on curetting biopsy, polypectomy with no residual tumour on subsequent resection | Clinical/  Pathological | Record TNM-T=1a and continue to Table 16 to derive RD-Stage | IA |
| **1b** | Tumour invading ≥50% of the myometrium | Use “1b” in case of:   - reported as invading 50% or more of the myometrium - endocervical glandular invasion WITH invasion 50% or more of the myometrium | Clinical/  Pathological | Record TNM-T=1b and continue to Table 16 to derive RD-Stage | IB |
| **2** | Tumour invading the stromal connective tissue of the cervix but not extending beyond the uterus | Use “2” in case of:   - reported as T=2 - reported as involving cervix uteri, NOS, but not beyond the uterus - reported as cervical stromal invasion | Clinical/  Pathological | Record TNM-T=2 and continue to Table 16 to derive RD-Stage | II |
| **3** | Tumour involving serosa, adnexa, vagina, or parametrium | Use “3” in case of:   - reported T=3 - reported as a tumour with extrauterine extension, NOS - reported extension or metastases to the pelvis, NOS | Clinical/Pathological | Record TNM-T=3 and continue to Table 16 to derive RD-Stage | III |
| **3a** | Tumour involving the serosa and/or adnexa | Use “3a” in case of:   - reported direct extension or metastases to tunica serosa (visceral serosa of corpus uteri) - reported direct extension or metastases to adnexa (ovary/ies, fallopian tube/s, broad, round and/or uterosacral ligaments) | Clinical/Pathological | Record TNM-T=3a and continue to Table 16 to derive RD-Stage | IIIA |
| **3b** | Tumour involving of the vagina or parametrium | Use “3b” in case of reported:   - as clinically described as “frozen pelvis” - direct extension or metastases to parametrium - direct extension or metastases to vagina - direct extension or metastases to pelvic serosa (visceral peritoneum of pelvic organs, excluding the serosa of corpus uteri) - direct extension or metastases to pelvic wall - extension to vulva and/or ureter - extension to bladder wall, excluding bladder mucosa - extension rectal wall, excluding rectal mucosa | Clinical/Pathological | Record TNM-T=3b and continue to Table 16 to derive RD-Stage | IIIB |
| **4** | Tumour invading bladder mucosa and/or bowel mucosa | Use “4” in case of reported:   - extension to bladder mucosa (excluding the bullous oedema) - extension to cul de sac (pouch of Douglas) - extension to small intestine - extension to bowel mucosa, NOS - as FIGO IVA or T=4 | Clinical/Pathological | Record TNM T=4a  **and**  **RD-Stage=4A**  (Staging completed) | **IVA**  (Staging completed) |

# Supplementary material 2. Diagnostic and treatment pathway- endometrial cancer


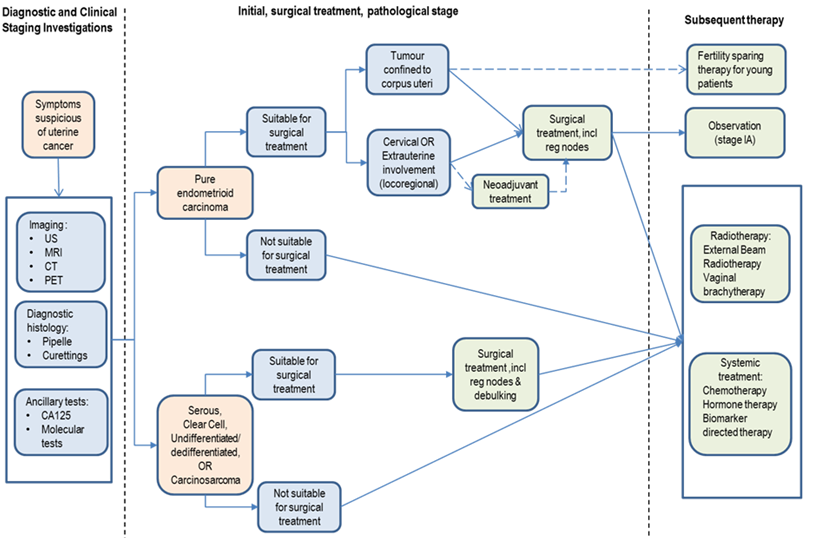

Supplement: Supplementary file 1 — Addditional file 1: Supplementary material 1. Staging rules for endometrial cancer. Supplementary material 2. Diagnostic and treatment pathway- endometrial cancer. [file 12885_2023_11615_MOESM1_ESM.docx]
